# Pyrodiversity is the coupling of biodiversity and fire regimes in food webs

**DOI:** 10.1098/rstb.2015.0169

**Published:** 2016-06-05

**Authors:** David M. J. S. Bowman, George L. W. Perry, Steve I. Higgins, Chris N. Johnson, Samuel D. Fuhlendorf, Brett P. Murphy

**Affiliations:** 1 School of Biological Sciences, University of Tasmania, Private Bag 55, Hobart, Tasmania, Australia; 2 School of Environment, University of Auckland, Private Bag 92019, Auckland, New Zealand; 3 Department of Botany, University of Otago, PO Box 56, Dunedin, New Zealand; 4 Natural Resource Ecology and Management, Oklahoma State University, Stillwater, Oklahoma, USA; 5 Research Institute for the Environment and Livelihoods, Charles Darwin University, Darwin, Northern Territory, Australia

**Keywords:** anthropogenic burning, ecosystem engineer, feedbacks, landscape fire, pyrogeography, trophic interactions

## Abstract

Fire positively and negatively affects food webs across all trophic levels and guilds and influences a range of ecological processes that reinforce fire regimes, such as nutrient cycling and soil development, plant regeneration and growth, plant community assembly and dynamics, herbivory and predation. Thus we argue that rather than merely describing spatio-temporal patterns of fire regimes, pyrodiversity must be understood in terms of feedbacks between fire regimes, biodiversity and ecological processes. Humans shape pyrodiversity both directly, by manipulating the intensity, severity, frequency and extent of fires, and indirectly, by influencing the abundance and distribution of various trophic guilds through hunting and husbandry of animals, and introduction and cultivation of plant species. Conceptualizing landscape fire as deeply embedded in food webs suggests that the restoration of degraded ecosystems requires the simultaneous careful management of fire regimes and native and invasive plants and animals, and may include introducing new vertebrates to compensate for extinctions that occurred in the recent and more distant past.

This article is part of the themed issue ‘The interaction of fire and mankind’.

## 1. Introduction

Human manipulation of landscape fires, whether deliberate or accidental, is a powerful ecological force that can influence the conservation of biodiversity and the provision of ecosystem services, and positively or negatively affect the risk of economically disruptive fires. Nonetheless, there remains substantial discussion and disagreement among fire managers, ecologists and conservation biologists over how best to achieve ecologically and economically sustainable fire management. This debate reflects the myriad competing objectives of fire management and the social values that influence them, combined with the complexity and uncertainties inherent in fire ecology. An example of these issues and concerns is the ‘pyrodiversity begets biodiversity’ hypothesis [1]—the idea that humans can promote biodiversity through the manipulation of the spatio-temporal component of fire regimes.

Martin & Sapsis [1] first introduced the term ‘pyrodiversity’ in their exploration of the biodiversity consequences of the transition from Native American fire management to twentieth-century fire suppression by government agencies. They characterized this transition as a shift from a pattern of anthropogenic burning that created and maintained fine-grained habitat mosaics, to one that reduced fire-induced heterogeneity in the landscape. This shift was driven by changes in the spatial extent (small to large), frequency (frequent to infrequent), seasonality (increase in summer) and severity (low to high) of fires. Martin & Sapsis [1] suggested that these changes reduce ‘pyrodiversity’ with accompanying losses of biodiversity, and recommended the implementation of heterogeneous fire regimes, tailored to suit particular environments and taxa, to conserve biodiversity.

**Figure 1.**
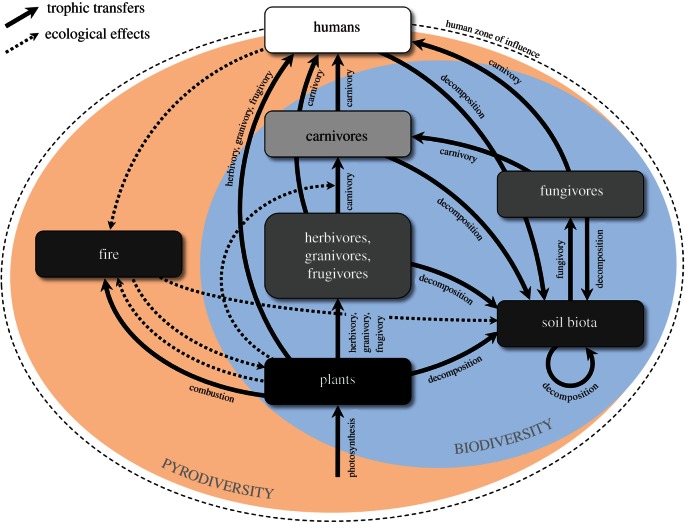
A conceptual overview of pyrodiversity, showing how fire regimes are embedded in food webs. The solid black lines indicate trophic transfers of carbon, analogous to the conventional links in a food web. Hence, landscape fire acts as a ‘consumer’ of plant biomass. However, fire has a range of other ecological effects (shown by dashed lines) on trophic processes, either directly, or indirectly (e.g. by modifying vegetation structure), which may facilitate predation. The shading of the components of the food web indicates the intensity of the biological refinement of organic carbon (quality), such as the carbon-to-nitrogen ratio. Plants, soil biota and fire are shaded black, indicating they produce and use low-quality carbon. The carbon quality is assumed to increase through the food chain with humans being shaded white, indicating they use the highest-quality carbon. Humans are assumed to directly influence all elements of the system (e.g. lighting and suppressing fire, harvesting animals and plants), and anthropogenic influences have now become predominate due to global climate change. Although humans are assumed to strongly influence pyrodiversity, it can still exist in their absence.

The distinction between the fire regime concept and pyrodiversity, and the linkage between biodiversity and pyrodiversity proposed by Martin & Sapsis [1], has resulted in ongoing debate and confusion [2–7]. These debates highlight many of the core intellectual and technical challenges in fire ecology and pyrogeography, including how fire regimes are defined and measured, how landscape fire history shapes ecosystems, and understanding how the coupling between humans and landscape fire has shaped ecosystems through time [8]. Here, we argue that landscape fire is an integral, albeit biophysically unique, component of food webs that connects fire regimes and biological diversity across trophic levels, including humans, the only organism to directly manipulate landscape fires: thus, we define pyrodiversity as the outcome of the trophic interactions and feedbacks between fire regimes, biodiversity and ecological processes (figure 1). It is important to note that some types of pyrodiversity are not reliant on human influence, whereas others, such as grasslands embedded in forest [9], are human artefacts.

Our conceptualization of pyrodiversity extends the view that landscape fire is a ‘global herbivore’, competing with other herbivores for fuel [10], seeing some species as having a metaphoric symbiotic and/or co-evolved relationship with fire. We suggest there is a spectrum of ecological states generated by landscape fire, each associated with a range of biodiversity conditions; some types of pyrodiversity emerge from ecologically degraded systems, whereas others enhance biodiversity. This conceptualization of pyrodiversity resonates with alternative stable-state theory, in which changes in fire, herbivory, or both, can cause rapid shifts between ecosystem states [11], the most iconic examples being the invasion of overgrazed rangelands by woody plants, the invasive grass–fire cycle [12], and the control of rainforest–savanna boundaries [13,14].

**Figure 2.**
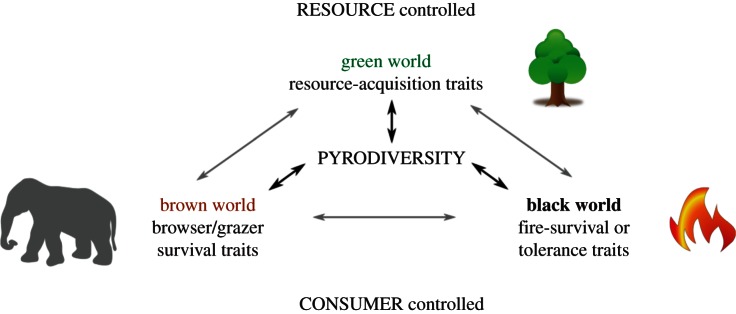
The trophic bounding of pyrodiversity due to the interplay of ecological conditions where biomass is predominantly constrained by fire, herbivores or resources. Adapted from Bond [16].

**Figure 3.**
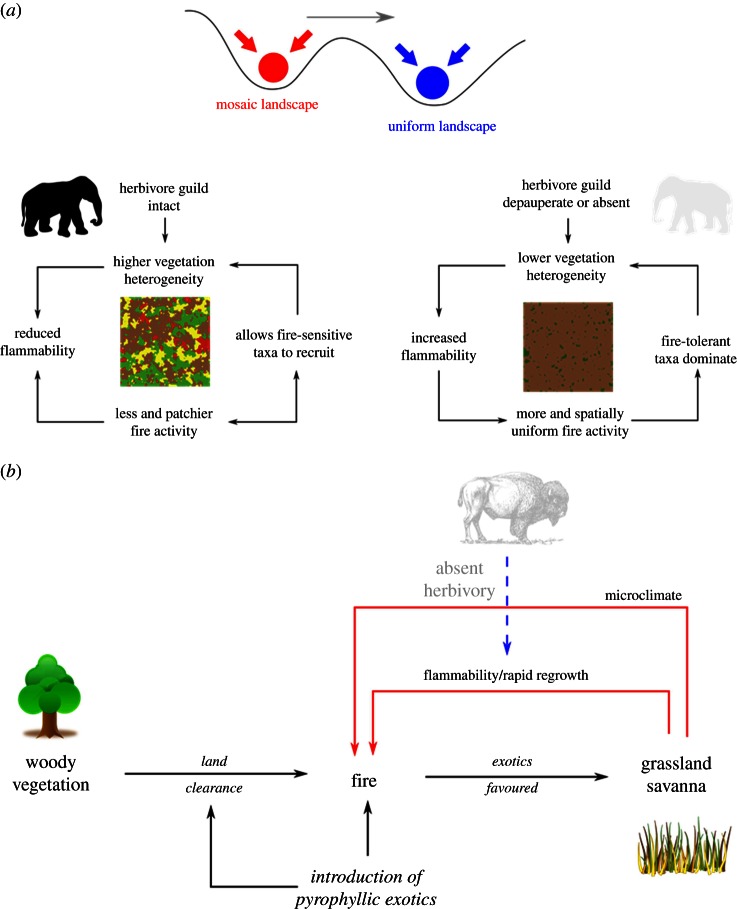
(*a*) A conceptual model of how megafaunal extinctions and altered fire regimes result in a switch in pyrodiversity. (*b*) The grass–fire cycle as an example of how the loss of consumer control can alter pyrodiversity (blue and red arrows reflect positive and negative feedbacks, respectively). (*a*) Redrawn from Bowman *et al.* [11] and (*b*) from D'Antonio & Vitousek [12].

The idea that fire modulates food webs has been anticipated by some authors [15]. For instance, Bond [16] suggested that the global distribution of vegetation may reflect the complex interplay between herbivores, environmental constraints and fire (figure 2), resulting in ‘black worlds’ where fire is the predominant constraint on biomass, ‘brown worlds’ where biomass is primarily regulated by herbivores, or ‘green worlds’ with biomass principally shaped by bottom-up resource constraints (climate and soils). Our conceptualization of pyrodiversity lies between Bond's [16] idealized black, brown and green worlds (figure 2) because top-down control by herbivores and bottom-up resource limitation together shape fire regimes and vegetation patterns [14]. Bond ([16], p. 264) noted that ‘it is intriguing to ask whether more of the world has become ‘black’ since extirpation/extinction of the megafauna’ (figure 3*a*). Applying a similar logic, we can reconceptualize the grass–fire cycle, which can degrade ecosystems following the introduction of invasive flammable grasses [12], as the absence of an accompanying coevolved herbivore to ‘compete’ with fire for grass biomass [17] (figure 3*b*).

To develop our view of pyrodiversity as an emergent property of fire embedded in food webs, we: (i) consider how this idea relates to the fire regime concept; (ii) review the correlative and mechanistic evidence for and against the importance of spatio-temporal fire patterns on biodiversity and how this influences ecological processes; and (iii) outline the implications of our argument for the management of ecosystems. Our focus is on ecosystems that have not been drastically transformed by land clearance; hence, most of our examples come from Australia, Africa and the western USA. Nonetheless, we believe our argument can be extended and applied to all ecosystems where fire was, is or potentially will be a key ecological disturbance.

## 2. Fire regimes and pyrodiversity

The term ‘fire regime’ captures the multi-dimensional nature of landscape fire [18]. Key characteristics of a fire regime include fire intensity, the time interval between fires, the spatial pattern of fires (size, shape), type of combustion (flaming versus smouldering), and the biogeochemical impacts that shape soils and vegetation [19]. Fire regimes filter biotas, selecting adaptations to tolerate and arguably even promote fire, reinforcing the tendency for a given pattern of fire to recur [20–23]. Even though the fire regime is a powerful organizing principle in fire ecology, it has proved remarkably difficult to operationalize as a metric that can be spatio-temporally analysed [24,25]. This problem has been summarized by Krebs *et al.* ([25], p. 61), who wrote that ‘in a complex process like fire, that involves temporal cascades, interactions and feedbacks, every cause is also an effect, every effect may be a causal variable, and no variable is truly independent. Any selection of the variables of the FR [fire regime] is therefore questionable and implies a significant degree of subjectivity’. It is in this complex intellectual milieu that the pyrodiversity concept is situated.

A narrow, trophically flat interpretation of pyrodiversity focuses exclusively on the spatio-temporal patterns of a fire regime. To empirically validate this comparatively simple definition, we need accurate time-series data to reveal the ‘invisible’ mosaic created by past fire events that interact with the visible mosaic created by the most recent fire event [26] (electronic supplementary material, figure S1). A range of techniques can be used to reconstruct spatio-temporal fire *pattern*, and each has constraints affecting the scale, accuracy and time-depth of historical reconstructions [27–30]. These reconstructions are often synthesized by a small number of static indices describing spatial or temporal patterns of fire activity or the landscape patterns arising from it (e.g. mean fire size, habitat diversity, fire return interval). Whether such metrics are sufficient to characterize landscape pattern and interactions between pattern and process is debatable [18,31]. Our conceptualization of pyrodiversity is not reducible to a single index, because it is hierarchical and multi-dimensional, requiring simultaneous consideration of both landscape *pattern* and ecological *process*, as is inherent in the interaction between fire regimes and biodiversity (figure 1). As outlined below, this conceptualization of pyrodiversity affects how it is studied and tested.

## 3. Does pyrodiversity beget biodiversity?

Martin & Sapsis's [1] seminal paper has stimulated an ongoing debate in conservation biology, over their ‘pyrodiversity begets biodiversity’ hypothesis and, by extension, the relevance of the fire management paradigm known as ‘patch mosaic burning’, which seeks to create and maintain spatio-temporal habitat heterogeneity in order to promote biodiversity [2,32]. Several researchers have investigated the pyrodiversity–biodiversity hypothesis by narrowly defining pyrodiversity as the spatio-temporal heterogeneity of landscape fire activity. For instance, a major research project in southeastern Australia's semi-arid eucalypt shrublands—known locally as ‘mallee’—found no consistent positive relationship between the Shannon–Weiner diversity of post-fire age-classes in the local area (2-km radius) and the abundance of individual species or species richness, among small mammals [3], birds [5] and reptiles [4]. This research concluded that the pyrodiversity–biodiversity hypothesis was not supported. However, this correlative ‘natural experiment’ has a number of limitations, including: (i) the failure to control for the ‘size, shape and interspersion of patches with differing fire histories, amount of ecotone habitat’ [5]; (ii) the assumption that the snap-shot surveys sampled landscapes that originally had similar faunal distributions and equivalent disturbance histories embedded in the ‘invisible’ burn mosaic [33]; and (iii) fundamental limits to the ability of simple metrics to capture the complexity of the spatial processes and interactions underpinning pyrodiversity [18,31].

The issue of the realism and sufficiency of the indices used to characterize fire regimes also affects experiments designed to test the pyrodiversity–biodiversity hypothesis. For example, in northern Australia's Kakadu National Park, where large homogeneous fires are implicated in biodiversity declines, Griffiths *et al.* [7] concluded that fire frequency has a far greater effect on populations of small mammals than fire size [34]. Their conclusion is derived from the outcomes of spatially explicit population models of four small-mammal species, built using data from a landscape-level fire experiment. However, Russell-Smith *et al.* [35] criticized this work as presenting grossly unrealistic scenarios—namely that the experimental fires imposed under the modelled ‘mosaic’ scenario were an order of magnitude larger than those typically experienced in Kakadu (15–20 km^2^ versus 1.2–4 km^2^, respectively). Hence, the ‘mosaic’ scenario of Griffiths *et al.* [7] does not even closely approximate the fine-grained mosaic advocated to conserve small mammals in Kakadu (i.e. less than 1 km^2^ [34,36]).

Simple, one-way statistical linkages between biodiversity surrogates and fire regimes are unlikely to identify crucial feedbacks between spatio-temporal patterns of burning and trophic interactions, because the direct impacts of such feedbacks reveal themselves on a variety of time-scales, and because direct impacts of fire on biodiversity may be nonlinear or conditional on other covariates [11]. Some experimental studies of pyrodiversity have focused on eusocial insects (e.g. ants and termites) [6,37,38], yet these species-rich communal organisms are possibly better buffered against changes in fire regimes than vertebrates, so there needs to be caution in extrapolating their response to fire to the entire biota. Rather, recognition of the trophic linkages between fire and the ecosystem as a whole demands detailed ecological studies to reveal mechanistic links between spatio-temporal mosaics of fire and particular species and species guilds.

Despite the weaknesses of correlative studies of the relationships between pyrodiversity and biodiversity, a consistent finding is the importance of relatively long-unburnt habitat for birds [5,39] and, to a lesser extent, mammals [3,40] and reptiles [4]. This agrees with the finding of Bradstock *et al.* [26], who used a simulation model to show that populations of the threatened ground-dwelling bird, malleefowl (*Leipoa ocellata*), could be sustained by a regime of small patchy fires. Kelly *et al.* [40] used decision theory to identify the ‘optimal’ fire regime for biodiversity conservation in the southeastern Australian mallee and found that vertebrate species diversity is likely to be maximized by a mix of early, middle and late successional vegetation, albeit not in equal proportions. Such heterogeneity is most likely to arise if the prime management objective is the creation and maintenance of fine-grained fire mosaics to ensure the persistence of long-unburnt habitats [26,41,42], which can be critical for many species.

## 4. Evidence of trophic linkages with fire

**Figure 4.**
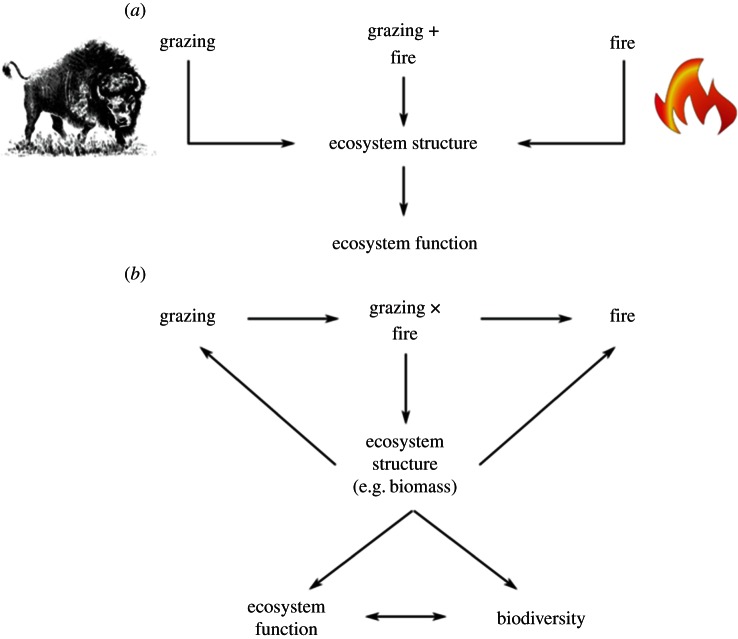
Conceptual model of the interaction between grazing and fire mosaics that drive ‘pyric herbivory’, with flow-on effects on biodiversity and ecosystem function. Adapted from Fuhlendorf *et al.* [43].

Studies of individual taxa illuminate the reciprocal relationships between biodiversity, ecosystem processes and the patterns generated by fire regimes. A good example of this is the ‘pyric herbivory’ concept that demonstrates the coupling between spatio-temporal fire patterns and grazing activity [43–45]. For example, bison (*Bison bison*) can reinforce fine-grained fire mosaics in North American tall-grass prairie ecosystems as their grazing reduces biomass and alters local species composition. This effect, in turn, reduces grazing pressure on the most palatable species because herbivores consume a broader range of species [43] (figure 4). The concentration of dung and urine produced by feeding animals further reinforces these biological effects. The resultant changes to vegetation structure affect the passage and intensity of subsequent fires, again reinforcing the fire mosaic. In North America, the pyric herbivory dynamic has a positive effect on the diversity of invertebrates [46–48], small mammals [44], birds [33,49], and productivity and behaviour of large native and domestic ungulates [45,50].

The interactions and feedbacks that create pyric herbivory are ecologically highly context-dependent in how they influence fire size and frequency, and their effects on biomass and fuel flammability [51–54]. This context dependence is clearly illustrated by Archibald *et al.* [51] in South African savannas. They demonstrated that frequent large fires can eliminate patches of grazing lawn, composed of short grazing-tolerant grasses that are embedded in tall, fire-dependent bunch grasslands. The mechanism driving the loss of grazing lawns is a reduction in the local intensity of grazing as large herbivores are enticed into surrounding burnt areas with resprouting grass. This mechanism depends upon both grazer density and biomass growth rate such that grazing lawns are less affected by landscape fire heterogeneity where there is high grazing pressure or low rainfall/productivity or *vice versa* [51]. In contrast to the South African dynamic, grazing lawns can become established and persist in some Tasmanian tussock grasslands following fire and subsequent intense herbivory by marsupials [53] (electronic supplementary material, figure S2).

The ecological interactions between small digging mammals and fire regimes also highlight the complex interplay of fire in food webs inherent in our conceptualization of pyrodiversity. Animals that dig for their food, and so disturb and turnover soil and generate micro-topographic variation in the form of foraging pits, have a broad range of ecological effects (electronic supplementary material, figure S3). These effects include increasing rates of organic matter decomposition, and thus nutrient cycling and soil formation, promoting water infiltration into soil, and creating safe sites for seed germination. Combined, these effects can increase ecosystem-level diversity and productivity [55]. One of the best examples of the coupling of digging animals with fire regimes involves the spreading of spores from ectomycorrhizal fungi specialized for *Eucalyptus* host trees. Johnson [56] found that sporocarp production was stimulated by fire, and that this caused a localized increase in the abundance of a mycophagous marsupial, the eastern bettong (*Bettongia gaimardi*). These animals dispersed the spores of ectomycorrhizal fungi, thereby facilitating the establishment of the mycorrhizal association in vegetation regenerating after fire. Digging animals may also interact with landscape fires by altering the amount and structure of fuel loads. For example, Nugent *et al.* [57] provide evidence that the superb lyrebird (*Menura novaehollandiae*), which forages by turning over large quantities of litter in southeastern Australian *Eucalyptus* forests, suppresses forest flammability by reducing connectivity of fine fuels and enhancing their decomposition. Further, Nugent *et al.* [57] found that when these ecosystem engineers are eliminated from severely burnt forests, there is an increase in the risk of subsequent fires.

In Australia, the specialist fossorial (digger) guild has suffered disproportionate extinction rates, raising concerns that there will be significant ecological transformations associated with loss of critical links in food webs [55,58]. If extensive fires remove ground cover, predation pressure on small mammals (such as diggers) may increase [59,60]. For example, McGregor *et al.* [61] attached video cameras with global positioning systems to collars on feral cats in northern Australian savannas and demonstrated sharp differences in hunting success (17% versus 70%) between micro-habitats with and without the refugia provided by dense grass and rocky terrain.

High fire frequencies often disadvantage late-successional tree species that produce large fruits, with direct and indirect effects on frugivores driving pyrodiversity state change (electronic supplementary material, figure S4). This is illustrated by Perry *et al.* [62], who described a suite of complex interactions between novel fire regimes, the decline of indigenous frugivorous birds, invasive pyrophyllic plants and exotic seed predators (rodents) in northern New Zealand, in an environment where fire was exceptionally rare before human colonization. In these landscapes, anthropogenic fire has reduced forest to small remnant patches and succession has been almost completely halted by a combination of seed predation and lack of dispersal. This slowed succession in turn makes the landscape more flammable for longer periods and provides a window for fire-tolerant and fire-promoting invasive plants to capture recently disturbed sites and increase flammability [63]. It seems likely that fire and exotic seed predators interact to divert successional trajectories in other Pacific islands (e.g. Hawaii [64]).

## 5. Fire management and pyrodiversity

There is increasing recognition by anthropologists, environmental historians and pyrogeographers of the positive and negative effects of human use of fire on ecosystems and biodiversity resulting in abrupt changes to pyrodiversity. For example, palaeoecological reconstructions show interrelated changes to fire regimes, vegetation type (including inferred structure) and food webs following the human-induced extinction of large body-mass animals in the Late Quaternary [65,66]. These studies suggest that large browsing animals created mosaics of open and closed vegetation, and that when humans caused their extinction these mosaics were lost due to accumulation of biomass that fuelled more severe fires [58,67] (figure 3*a*). This model is supported by evidence from the eastern USA, Australia and Madagascar, showing that declines in dung fungal spores (a proxy for megafaunal populations) were followed by an increase in charcoal abundance (signalling increased and more extensive landscape fire) and then a shift to more fire-tolerant vegetation [68–70].

In contrast to the ecological upheavals that followed the megafaunal extinctions, skillful fire management by indigenous peoples in the recent past created landscape fire patterns at a much finer grain than occurs under natural ignition regimes [41,71–76]. These studies suggest a wide variety of utilitarian motives for the creation of fire mosaics, including increasing the abundance of game using the principle of pyric herbivory, reducing the risk of large uncontrolled fires and generally making landscapes more suitable for humans [41,50,77]. Irrespective of motivation, or even explicit awareness of the ecological outcomes, there is considerable evidence that this activity promotes biodiversity [71,78]. For instance, a heuristic simulation modelling exercise by Trauernicht *et al.* [41] compared mosaics of numerous small fires with mosaics of few large fires (though occupying the same total area), demonstrating that a finer-grained mosaic produces more patches of long-unburnt habitat, which provide refugia for fire-sensitive plants and animals across the landscape, such as the fire-sensitive obligate-seeding tree (*Callitris intratropica*) that persists in very fire-prone Australian tropical savanna under the management of the Gunei people. This Aboriginal group use fire for a wide variety of purposes that indirectly benefit many species, although their patch burning on drainage lines in the late dry season is explicitly designed to promote local abundance of kangaroos (*Macropus* spp.) [79]; one elder explained that ‘fire is for kangaroos’ [80]. Likewise, Codding *et al.* [81] found *Macropus robustus* abundance was greatest in desert habitats actively burnt by Aboriginal people, creating fine-grained pyrodiversity.

**Figure 5.**
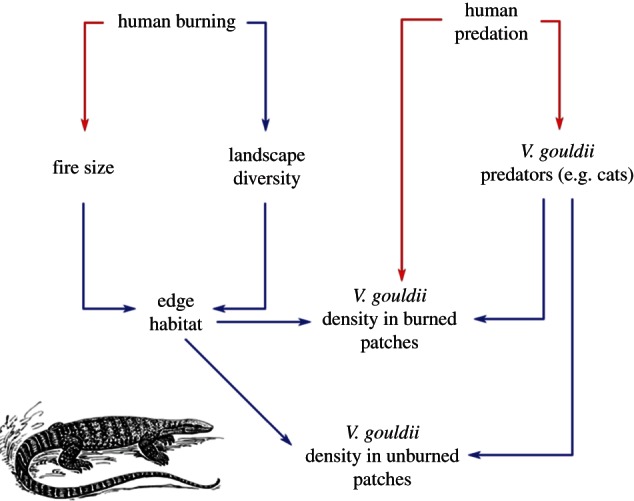
Conceptual model of the effect of indigenous fire management on food-webs and the abundance of a key prey item, the monitor lizard *Varanus gouldii*, in the Western Desert of Australia. Red and blue arrows show negative and positive feedbacks/interactions, respectively. Adapted from Bird *et al.* [82].

Perhaps the most striking example of human-induced pyrodiversity is described by Bird *et al.* [82], who demonstrated that, paradoxically, the greatest abundance of the large lizard *Varanus gouldii* occurs where Aboriginal hunting is most intense (figure 5). They explained this as a consequence of the fine-grained pyrodiversity created by Aboriginal hunting fires, combined with human predation of feral cats, and describe it as ‘dreamtime logic’, where fire management improved habitats for key harvested animal species. Bird *et al.* [83] suggest that Aboriginal hunters should be considered ‘trophic facilitators’ because of their creation of habitat mosaics that appear critical for the persistence of small mammals. Another important effect of Aboriginal patch burning is buffering against large fires driven by inter-annual climate variability such as that related to the El Niño Southern Oscillation [83]. Bird *et al.* [83] showed that in the absence of patch burning, lightning-ignited fires are orders of magnitude larger following seasons of high rainfall. Such buffering is important in environments where wildlife populations experience boom–bust cycles. In Australia, the effect of inter-annual climate variability has been amplified by the cessation of Aboriginal fire management in most areas. The resultant larger fires, combined with introduced prey (rabbits and black rats) and predators (cats and foxes) that irrupt under wet La Niña conditions, may trigger extinction cascades due to hyper-predation and the loss of the unburnt habitats critical for provision of food resources and shelter during dry El Niño conditions [84] (electronic supplementary material, figure S5).

The decline of granivorous birds in northern Australian savannas has been attributed, at least partly, to altered fire regimes, and the loss of fine-grained Aboriginal fire mosaics [85]. This hypothesis has been experimentally validated by a landscape-scale intervention in northern Australia by Legge *et al.* [86]. These authors demonstrated three granivorous finches in northern Australia suffered physiological stress under ‘extensive, intense fires, which homogenise the spatio-temporal variability’. Reduced fire frequency and increased extent of relatively long-unburnt (more than three years) vegetation significantly improved the condition of these birds, as the availability of grass seeds increased during the late dry and wet seasons. The breakdown of Aboriginal fire mosaics has also disadvantaged fruit trees and fruit-eating animals [87–89]. Such results provide support for management interventions such as burning in the early dry season when fuel moistures limit the spatial extent and intensity of fires, designed to increase fine-grained pyrodiversity in these savanna landscapes [90].

An excellent example of how human actions can change pyrodiversity both directly and indirectly concerns dry, low-elevation western USA ponderosa pine forests, where Martin & Sapsis [1] originally proposed the pyrodiversity–biodiversity nexus. These forests are believed to have evolved to tolerate frequent low-severity fires under a summer lightning fire regime [91]. Native American fire management probably increased the frequency of fires, creating parklands with a grassy understorey, through both patch burning and harvesting of wood and other fuels around permanent settlements such as Pueblo [92]. European colonization in the late eighteenth century disrupted indigenous fire management and changed fuels through overgrazing and logging, especially following the construction of railways in the ninteenth century [93]. In order to reduce large uncontrolled fires, a policy of total fire suppression was implemented in the early twentieth century. Reduced fire activity lead to a change in forest structure from open to closed understoreys densely stocked with *Pinus* saplings, resulting in infrequent, geographically large, high-severity crown fires that have disadvantaged some components of biodiversity [94]. New approaches to reduce the extent of these ‘megafires’ involve ecological restoration of fire regimes, with interventions including mechanical thinning of overstocked stands, and in localized cases the use of herbivores to return these forests to more open communities with a low-severity, surface fire regime [95,96].

Ecological restoration of pyrodiversity requires more than the reimposition of fire regimes if keystone taxa, such as herbivores that create grazing lawns, small digging animals that drive nutrient cycling, frugivores that disperse seeds and predators to regulate herbivores have been eliminated. By the same token, successful reintroduction of animal and plant species requires careful consideration of the restoration of appropriate fire (and other disturbance) regimes by manipulating fuel loads by harvesting fuels, clearing forests or introducing plant species, influencing grazing and browsing pressure, and active fire suppression [8]. Such ecological restoration programmes are increasingly important given the influence of humans on ecosystems across the globe, including disrupting fire regimes and altering food webs by deliberately or accidentally creating novel ecological assemblages.

## 6. Conclusion

We define pyrodiversity as the outcome of complex interactions and feedbacks between fire regimes, biodiversity and ecosystem effects (figure 1). This definition captures the interplay between landscape patterns and ecological processes. We see parallels with the delineation of biodiversity, which involves both enumeration of objects (e.g. genes, populations, species, ecosystems) and processes that shape these objects (e.g. natural selection, demography, population dynamics). Our conceptualization of pyrodiversity situates the fire regime concept in a trophic framework by extending the notion that fire is a ‘global herbivore’ to it being a broad-spectrum ‘ecological engineer’ with diverse trophic interactions, that in some cases has parallels with symbiosis and coevolution. Foundation writings in ecosystem theory have failed to adequately represent the trophic effects of fire. Indeed, fire is typically treated as a simple limiting factor along with soil nutrients [97]. The ‘thought experiment’ of Bond *et al.* [98] of a ‘world without fire’ has catalysed much recent activity showing how important fire can be in driving global vegetation patterns [99,100]. Ecosystem modellers have only recently begun to embrace the interactive effects of landscape fire, especially feedbacks between the biota and fire, in their thinking. However, ecosystem models have begun to represent the interactive effects of herbivory and fire [101,102], and fire behaviour models have started to consider herbivory in shaping landscape fire [103]. Each of these examples has taken very different approaches to modelling the fire–herbivory interaction. Scheiter & Higgins [104] found that fire, CO_2_ and herbivory interact strongly to shape vegetation structure globally, whereas other studies have suggested that the effect is far more localized [102,103]. However, given the nonlinear nature of the interactions and feedbacks inherent in our conceptualization of pyrodiversity, no single method of enquiry can be expected to disclose all of the underlying controls. Rather, integrated research—using ecological and historical narratives, statistical analysis, experimentation and modelling—is required to understand an environmental modulator like landscape fire [11,15].

Our conceptualization of pyrodiversity emphasizes the special ecological role played by humans—the only species able to directly and deliberately manipulate landscape fires through a variety of management actions [8]. This view demands that scientists and managers understand and interrogate the feedbacks between fire, vegetation and animals. Holistic studies, targeting trophic interactions and feedbacks, are required and we recommend against over-reliance on simple experimental or correlative study designs [11]. Such reimagining and reframing of fire as a modulator of trophic interactions opens up new ways of managing fire that involve manipulating wildlife and vegetation as much as directly altering fuel loads and ignition rates. This reimagining includes breaking the invasive grass–fire cycle using herbivores, replacing extinct megafauna to restore vegetation mosaics, and sustaining frugivore populations by re-establishing fruit trees in degraded habitats. Fundamentally, pyrodiversity shows that humans *are* a central actor in a web of interactions with fire, highlighting the wisdom in the adage that ‘fire is a good servant and a bad master’.

## 7. Meeting discussion

*Toddi Steelman* (University of Saskatchewan, Canada). If we need to manage for pyrodiverse patterns and processes, which patterns and processes do we manage for given the current patterns and processes in existence? What is the new goal?

*D.M.J.S.B.*: Understanding pyrodiversity as the emergent property of the interactions between biodiversity, fire regimes and ecological processes shapes the way we understand fire management, land management and restoration ecology. The question of management objectives, however, hinges on values because pyrodiversity is an ecological state that is neither ‘good' nor ‘bad'. Given the stress on the Earth system from anthropogenic impacts, particularly through climate change, declining biodiversity and disruption to ancient traditions of fire management, there is a need to manipulate pyrodiversity to achieve sustainable outcomes such as enhancing ecosystem services, reducing the risk of catastrophic fires and maximizing biodiversity. This may be achieved through the creation of novel ecosystems, ecological replacement of extinct keystone species or restoration and maintenance of historical fire regimes. Such interventions carry risk and demand monitoring and adaptive management. Management that ignores pyrodiversity through a narrow fixation on individual elements of fire regimes, specific biodiversity components and ecological process is unlikely to result in sustainable outcomes or meet management objectives.

## Supplementary Material

Supplementary figures
