# Supplementary material for: Pyrodiversity is the coupling of biodiversity and fire regimes in food webs
Source: Philos Trans R Soc Lond B Biol Sci. 2016 Jun 5;371(1696):20150169. doi: 10.1098/rstb.2015.0169 (PMC4874407; doi:10.1098/rstb.2015.0169)
Supplement: Supplementary figures [file rstb20150169supp1.pdf]

Bowman DMJS, Perry GLW, Higgins SI, Johnson CN, Fuhlendorf SD and Murphy BP 2016. Pyrodiversity is the coupling of biodiversity and fire regimes in food-webs. *Phil. Trans. R. Soc. B* **371**
doi: 10.1098/rstb.2015.0169

Supplementary figures

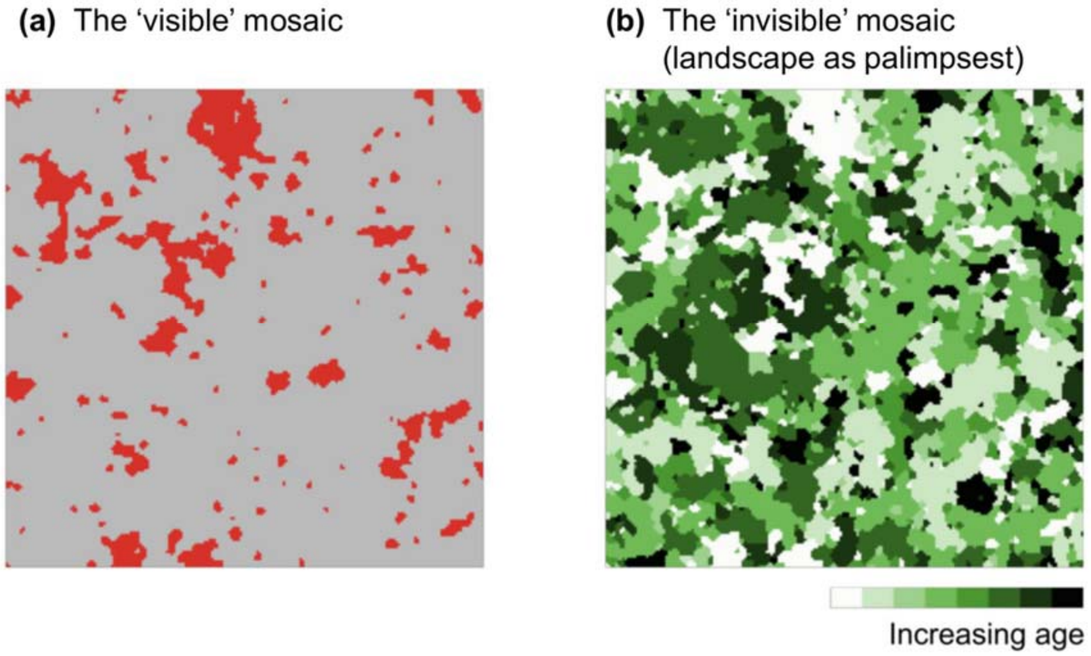

Figure S1. The visible and invisible mosaics that underpin conventional conceptualisations of pyrodiversity. A single fire creates spatial patterns in burnt areas (a), yet these patterns are shaped by past fires, and multiple fires create mosaic of habitats of differing ages since the last fire (b). Such patterns can have substantial ecological effects on biodiversity. Redrawn from Parr and Andersen [1].

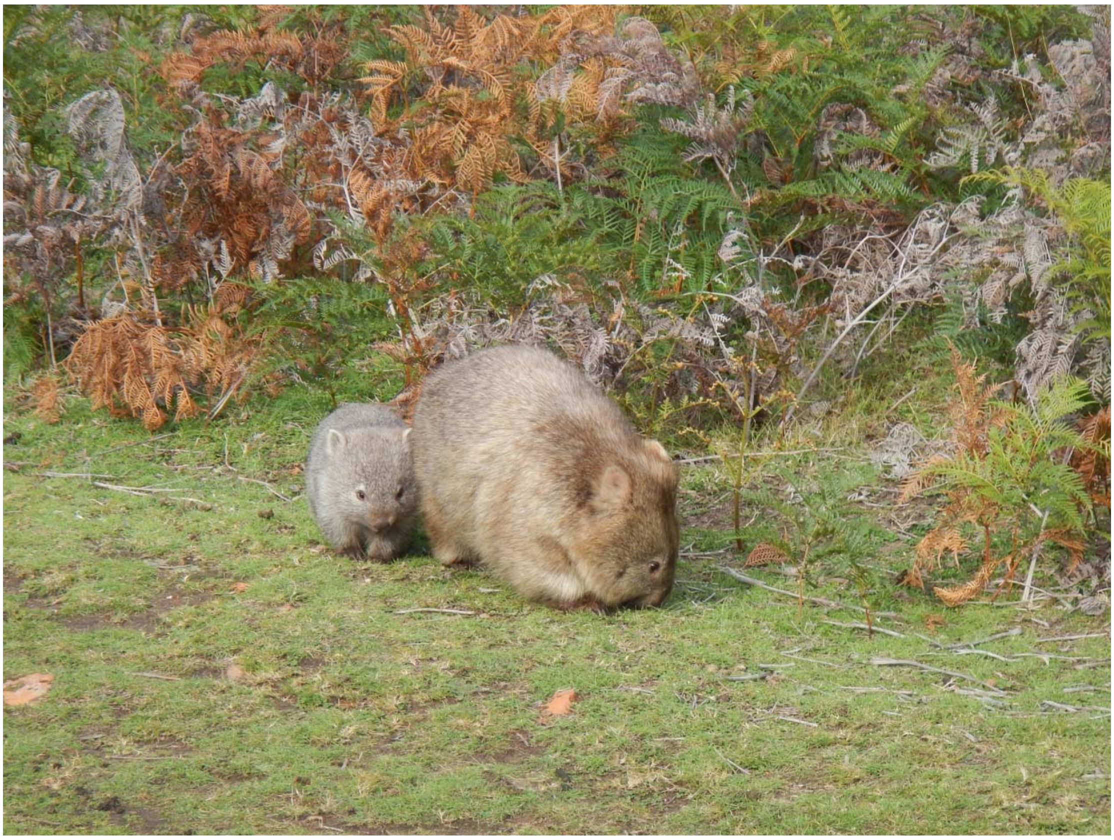

Figure S2. Sustained grazing by the common wombat (*Vombatus ursinus*) can maintain lawns in Tasmanian grasslands.

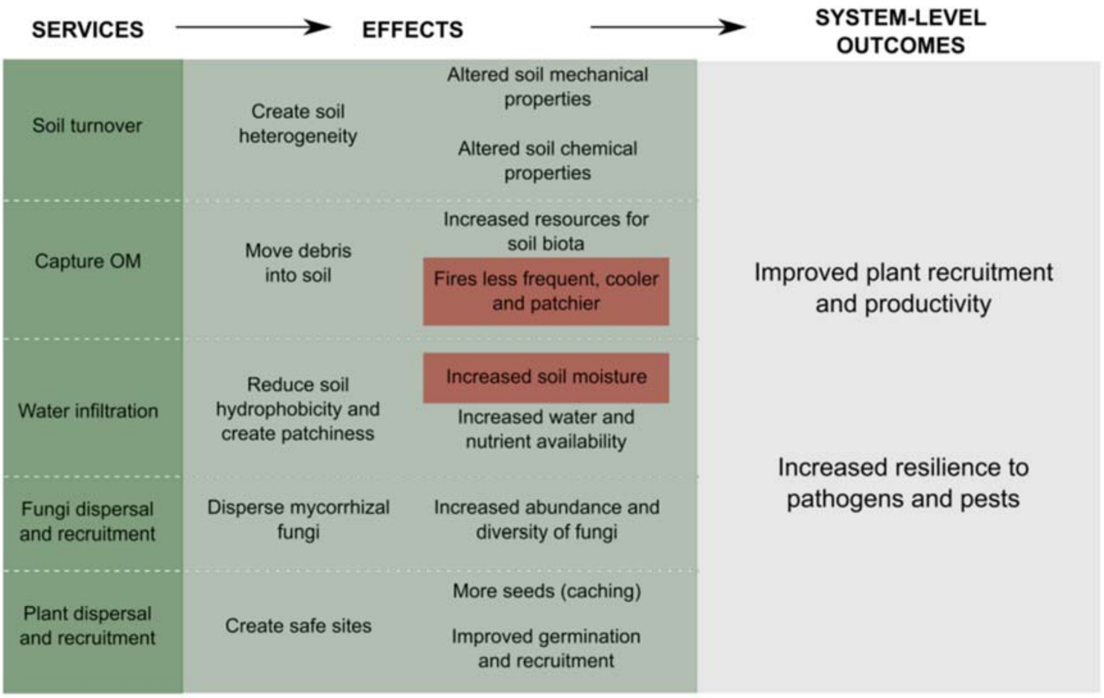

Figure S3. Ecological consequences of the loss of small digging animals in Australian ecosystems, a process that has been attributed to changes in the increased size and frequency of landscape burning following European colonisation. Orange boxes highlight direct effects on landscape fire. Redrawn from Fleming [2].

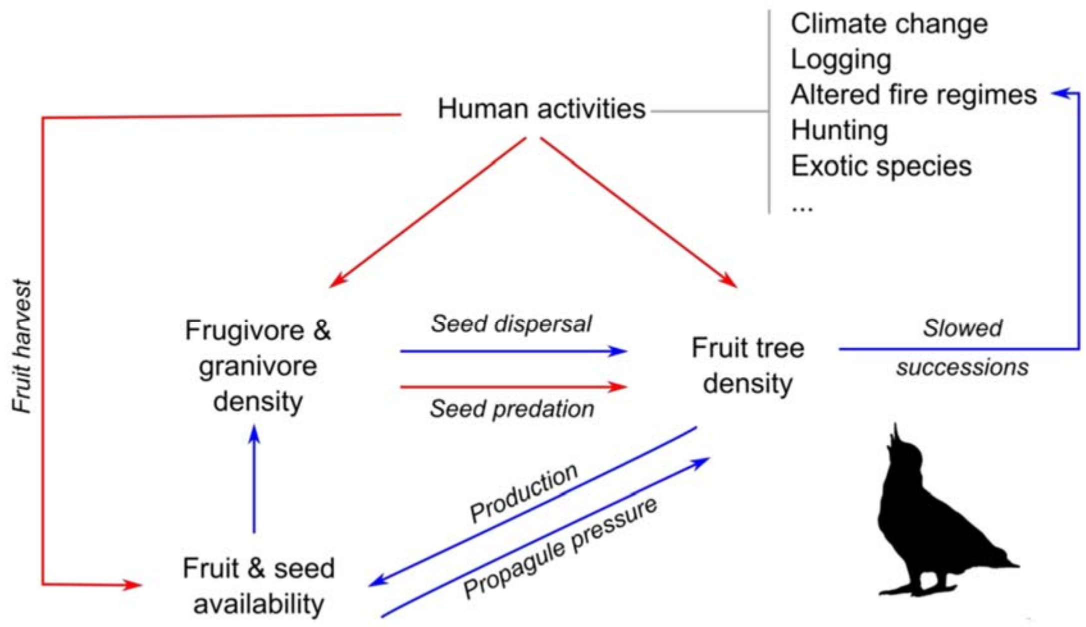

Figure S4. Conceptual model of the interaction of introduced plants, loss of frugivores and altered landscape fire activity driving land cover change and altered pyrodiversity. Red and blue arrows show negative and positive feedbacks/interactions, respectively. Redrawn from Brodie and Aslan [3].

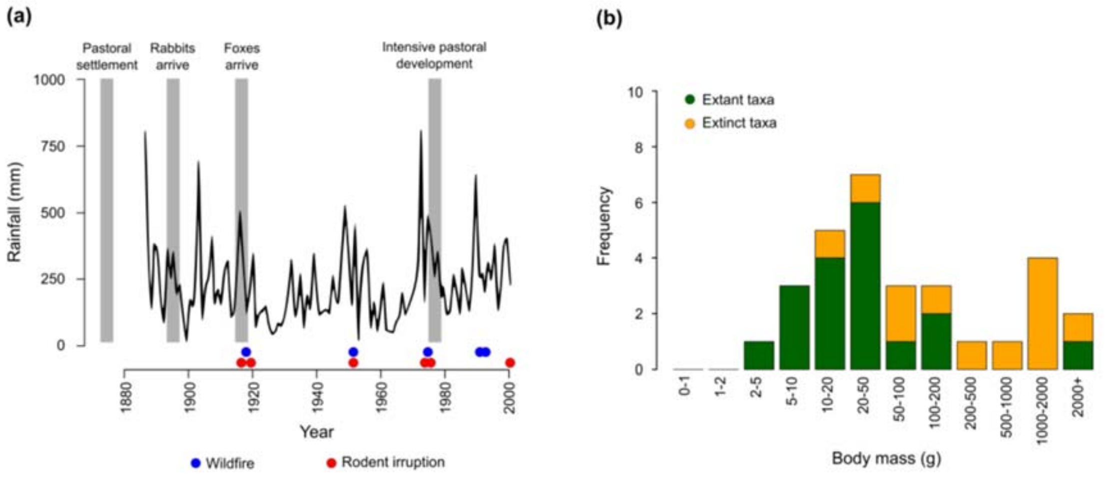

Figure S5. The effect of climate variability on pyrodiversity in the arid lands of Australia. (a) Temporal dynamics showing how changes to food-webs followed the introduction of herbivores and predators along with climate-driven large-scale fire activity. (b) The cascade of extinctions of small mammals driven by altered pyrodiversity. Redrawn from Letnic and Dickman [4].

References

1. Parr, C.L. & Andersen, A.N. 2006 Patch mosaic burning for biodiversity conservation: a critique of the pyrodiversity paradigm. *Conserv. Biol.* **20**, 1610-1619.

2. Fleming, P.A., Anderson, H., Prendergast, A.S., Bretz, M.R., Valentine, L.E. & Hardy, G.E.S. 2014 Is the loss of Australian digging mammals contributing to a deterioration in ecosystem function? *Mammal Rev.* **44**, 94-108.

3. Brodie, J.F. & Aslan, C.E. 2012 Halting regime shifts in floristically intact tropical forests deprived of their frugivores. *Restoration Ecol.* **20**, 153-157.

4. Letnic, M. & Dickman, C. 2006 Boom means bust: interactions between the El Niño/Southern Oscillation (ENSO), rainfall and the processes threatening mammal species in arid Australia. *Biodivers. Conserv.* **15**, 3847-3880.
